# The Airway Microbiota Signatures of Infection and Rejection in Lung Transplant Recipients

**DOI:** 10.1128/spectrum.00344-21

**Published:** 2022-04-13

**Authors:** Jin Su, Chun-xi Li, Hai-yue Liu, Qiao-yan Lian, Ao Chen, Zhi-xuan You, Kun Li, Yu-hang Cai, Yan-xia Lin, Jian-bing Pan, Guo-xia Zhang, Chun-rong Ju, Chang-xuan You, Jian-xing He

**Affiliations:** a Department of Respiratory and Critical Care Medicine, Chronic Airways Diseases Laboratory, Nanfang Hospital, Southern Medical University, Guangzhou, China; b Department of Clinical Laboratory, the First Affiliated Hospital of Xiamen University, Xiamen, China; c State Key Laboratory of Respiratory Disease, National Clinical Research Center for Respiratory Disease, Guangzhou Institute of Respiratory Health, the First Affiliated Hospital of Guangzhou Medical University, Guangzhou, China; d Nanshan School, Guangzhou Medical University, Guangzhou, China; e Institute of Antibody Engineering, School of Laboratory Medicine and Biotechnology, Southern Medical University, Guangzhou, China; f Hospital Infection-Control Department, Shenzhen University General Hospital, Shenzhen, China; g Department of Environmental Health, Guangdong Provincial Key Laboratory of Tropical Disease Research, School of Public Health, Southern Medical University, Guangzhou, China; h Department of Oncology, Medical Center for Overseas Patient, Nanfang Hospital, Southern Medical University, Guangzhou, China; Huazhong University of Science and Technology

**Keywords:** 16S rRNA, airway microbiota, infection, lung transplant, rejection

## Abstract

Infection and rejection are the two most common complications after lung transplantation (LT) and are associated with increased morbidity and mortality. We aimed to examine the association between the airway microbiota and infection and rejection in lung transplant recipients (LTRs). Here, we collected 181 sputum samples (event-free, *n* = 47; infection, *n* = 103; rejection, *n* = 31) from 59 LTRs, and performed 16S rRNA gene sequencing to analyze the airway microbiota. A significantly different airway microbiota was observed among event-free, infection and rejection recipients, including microbial diversity and community composition. Nineteen differential taxa were identified by linear discriminant analysis (LDA) effect size (LEfSe), with 6 bacterial genera, *Actinomyces, Rothia, Abiotrophia, Neisseria, Prevotella*, and *Leptotrichia* enriched in LTRs with rejection. Random forest analyses indicated that the combination of the 6 genera and procalcitonin (PCT) and T-lymphocyte levels showed area under the curve (AUC) values of 0.898, 0.919 and 0.895 to differentiate between event-free and infection recipients, event-free and rejection recipients, and infection and rejection recipients, respectively. In conclusion, our study compared the airway microbiota between LTRs with infection and acute rejection. The airway microbiota, especially combined with PCT and T-lymphocyte levels, showed satisfactory predictive efficiency in discriminating among clinically stable recipients and those with infection and acute rejection, suggesting that the airway microbiota can be a potential indicator to differentiate between infection and acute rejection after LT.

**IMPORTANCE** Survival after LT is limited compared with other solid organ transplantations mainly due to infection- and rejection-related complications. Differentiating infection from rejection is one of the most important challenges to face after LT. Recently, the airway microbiota has been reported to be associated with either infection or rejection of LTRs. However, fewer studies have investigated the relationship between airway microbiota together with infection and rejection of LTRs. Here, we conducted an airway microbial study of LTRs and analyzed the airway microbiota together with infection, acute rejection, and clinically stable recipients. We found different airway microbiota between infection and acute rejection and identify several genera associated with each outcome and constructed a model that incorporates airway microbiota and clinical parameters to predict outcome. This study highlighted that the airway microbiota was a potential indicator to differentiate between infection and acute rejection after LT.

## INTRODUCTION

Lung transplantation (LT) is the only therapeutic option for patients with end-stage lung disease. Although the short-term graft survival of lung transplant recipients (LTRs) has improved over decades, it is still limited compared with that of other solid organ transplant recipients (1). The low survival rate is predominantly due to infection- and rejection-related complications, which are the two major threats for LTRs in both early and long-term follow-up (1, 2). Severe acute rejection often occurs after LT, and various infections due to an immunosuppressed state and the unique anatomy and physiology of the transplanted lung also occur frequently (2). As a result, rejection and infection affect and interact with each other, and balancing rejection and infection is the major challenge of LT (3). Many studies have revealed the importance of the airway microbiota in local and systemic immunity, and airway microbiota dynamics play a very important role in the development and pathophysiology of respiratory diseases (4–6). This suggests that the airway microbiota may affect the immune response and therefore the balance between infection and rejection in LTRs.

Over the past decades, several studies have observed an altered airway microbiota in LTRs compared with that in healthy controls and pretransplant patients (7–9). For example, Charlson et al. found that LTRs have lower microbial richness and diversity but a higher bacterial burden in bronchoalveolar lavage fluid (BALF) than control subjects (7). Syed and colleagues demonstrated a similar alpha diversity and a distinct beta diversity of the airway microbiota between pre- and post-LT (8). In addition, Pseudomonadaceae, Enterobacteriaceae and Staphylococcaceae were enriched in LTRs, while Prevotellaceae, Veillonellaceae and Streptococcaceae were frequently detected in nontransplant individuals (9). Importantly, increasing evidence has indicated a close relationship between the airway microbiota and the disease progression and outcome of LTRs (10–12). In summary, investigation of the airway microbiota may improve our understanding of the mechanisms involved in allograft dysfunction and may suggest potential therapies to improve survival for LTRs.

In addition to the immunosuppressed state, LTRs are more susceptible to infection because of direct exposure to the external environment, a defective cough reflex and damaged mucociliary clearance compared with those in other organ transplant patients (2, 13). Respiratory infection is the main cause of death within the first year after LT, with bacteria being the most frequent cause (13, 14). A recent study suggested a distinct airway microbiota in LTRs between respiratory infection and colonization without respiratory infection (15), and loss of airway microbial diversity can increase the risk of infection (9). BALF neutrophilia was found to be associated with lower microbial diversity, indicating a correlation between the airway microbiota and infection after LT (16). Allograft rejection is another common complication after LT and is associated with increased morbidity and mortality (3). Previous studies have revealed a significant relationship between the airway microbiota and acute rejection. For example, low microbial diversity was associated with acute rejection (17), while microbiome phenotypes dominated by *Actinobacteria* reduced the risk of developing acute rejection (12). Overall, these studies indicated a close relationship between the airway microbiota and infection and rejection of LTRs.

The treatment of infection and acute rejection is completely different. The former needs antimicrobial therapy and the latter needs immunosuppressive therapy. If the patient is incorrectly diagnosed, the use of immunosuppressants will aggravate the existing infection, and unnecessary antibiotic treatment often has toxic side effects in patients with rejection only. Therefore, an early and correct diagnosis is the premise for effective treatment. However, it is sometimes difficult to clinically differentiate between acute rejection and infection, especially when patients have fever and nonspecific symptoms or signs. In addition, both posttransplant infection and acute rejection may be accompanied by similar clinical features, including cough, shortness of breath and radiological infiltrate, thus resulting in difficult differential diagnosis (18). Although clinicians try to monitor these two complications carefully, the diagnostic options are limited. The diagnosis of infection requires clinicians to identify the source and carry out pathogen specific tests. The pathological assessment of transbronchial biopsy specimens, which is the gold standard for the diagnosis of acute rejection after LT, is an invasive procedure with a high degree of variability and limited reliability (19). Considering the high morbidity and mortality of infection and rejection in LTRs and the bidirectional relationship between them, clinical decision-making depends on accurate diagnosis of both infection and rejection. Recent studies have revealed an association between the airway microbiota and infection or rejection after LT. However, fewer studies have investigated the specific microbial differences between infection and acute rejection recipients. Whether there are potential indicators to distinguish between infection and acute rejection in LTRs remains unknown. In this cross-sectional study, we analyzed the airway microbial profiles associated with infection and acute rejection in LTRs.

## RESULTS

**FIG 1:**
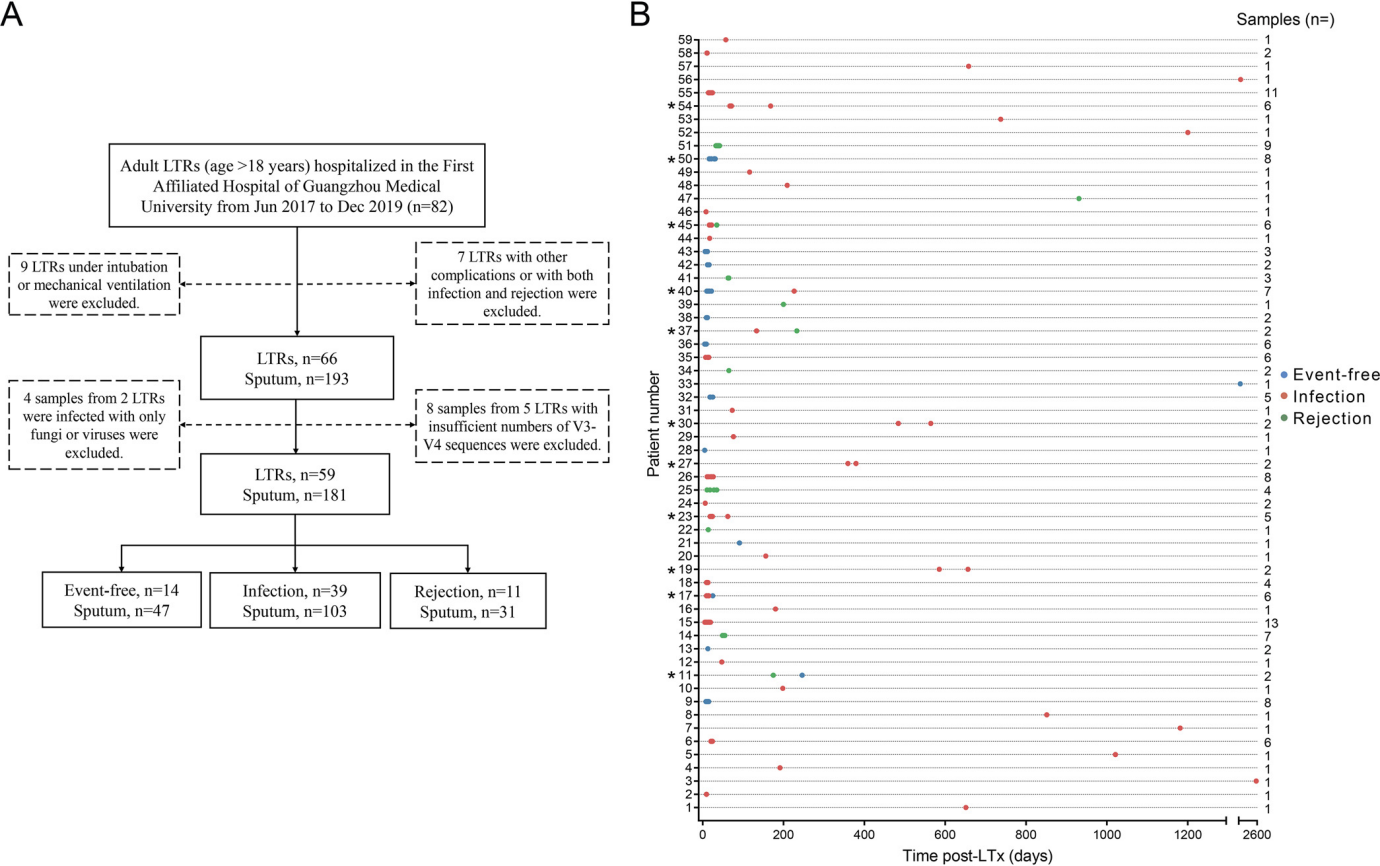
Information of patient recruitment and samples collection. (A) Study flow chart. (B) Dots represent sputum samples collected from each of the 59 patients after LT. Patients 1–18 had COPD, patients 19–50 had ILD, and patients 51–59 were diagnosed with other lung diseases. *Patients who were sampled during two hospitalizations and diagnosed with two clinical statuses (same or different).

After sequencing, a total of 181 sputum samples from 59 LTRs were included for subsequent analysis (Fig. 1). We divided the recipients into three groups according to the presence or absence of pulmonary infection and acute rejection at sampling: clinically stable (or event-free, *n* = 47) recipients, recipients with infection (*n* = 103), and recipients with rejection (*n* = 31). The clinical characteristics of the LTRs are presented in Table 1.

**TABLE 1:** Patient characteristics^*a*^

Characteristic	Total	Event-free	Infection	Rejection
Patients/samples	59/181	14/47	39/103	11/31
Sex (male)	49 (83.1%)	13 (92.9%)	31 (79.5%)	9 (81.8%)
Age, yrs (mean±SD)	57.2 ± 12.8	60.5 ± 13.1	57.0 ± 12.1	53.9 ± 15.7
Type of transplant				
Double	17 (28.8%)	3 (21.4%)	15 (38.5%)	1 (9.1%)
Single	41 (69.5%)	11 (78.6%)	24 (61.5%)	9 (81.8%)
Heart-lung transplant	1 (1.7%)	1 (7.1%)	0 (0.0%)	1 (9.1%)
Time posttransplant (days)	284.2 ± 484.6	51.6 ± 206.0	161.1 ± 366.7	87.3 ± 164.5
BMI (kg/m^2^)	20.4 ± 3.7	20.6 ± 3.2	19.8 ± 3.5	22.1 ± 4.0
History of smoking, yes	36 (61.0%)	11 (73.3%)	24 (54.5%)	7 (63.6%)
PGD grade	2.0 ± 1.1	1.6 ± 1.1	2.2 ± 1.0	4.7 ± 2.9
Pretransplant diagnosis				
COPD	18 (30.5%)	4 (28.6%)	14 (35.9%)	2 (18.2%)
ILD	32 (54.2%)	10 (71.4%)	17 (43.6%)	8 (72.7%)
Other	9 (15.3%)	0 (0.0%)	8 (20.5%)	1 (9.1%)
Laboratory parameters^*b*^				
PCT (ug L^-1)	0.2 ± 0.5	0.1 ± 0.1	0.3 ± 1.0	0.1 ± 0.1
Blood T lymphocyte (/UL)	298.0 ± 261.0	315.2 ± 236.1	450.3 ± 407.2	280.6 ± 142.6
Positive culture^*b*^^,^^*c*^				
Acinetobacter baumannii	24 (13.3%)	9 (19.1%)	15 (14.6%)	0 (0.0%)
Enterobacter sp.	2 (1.1%)	0 (0.0%)	2 (1.9%)	0 (0.0%)
*Enterococcus* sp.	14 (7.7%)	2 (4.3%)	12 (11.7%)	0 (0.0%)
Klebsiella pneumoniae	31 (17.1%)	14 (29.8%)	8 (7.8%)	9 (29.0%)
Pseudomonas aeruginosa	29 (16.0%)	0 (0.0%)	20 (19.4%)	9 (29.0%)
Staphylococcus sp.	27 (14.9%)	6 (12.8%)	20 (19.4%)	1 (3.2%)
Stenotrophomonas maltophilia	43 (23.8%)	12 (25.5%)	24 (23.3%)	7 (22.6%)
Haemophilus influenzae	5 (2.8%)	0 (0.0%)	5 (4.9%)	0 (0.0%)
Aspergillus sp.	15 (8.3%)	0 (0.0%)	15 (14.6%)	0 (0.0%)
*Candida* sp.	5 (2.8%)	0 (0.0%)	5 (4.9%)	0 (0.0%)
Blood CMV DNA	11 (6.1%)	0 (0.0%)	11 (10.7%)	0 (0.0%)
Antibiotics^*b*^				
Meropenem/Vancomycin	90 (49.7%)	20 (42.6%)	66 (64.1%)	4 (12.9%)
Piperacillin/Cefoperazone	72 (39.8%)	19 (40.4%)	34 (33.0%)	19 (61.3%)
TMP/SMX	29 (16.0%)	1 (2.1%)	24 (23.3%)	4 (12.9%)
Azithromycin	4 (2.2%)	1 (2.1%)	3 (3.0%)	0 (0.0%)
Immunosuppression^*b*^				
Glucocorticoid	181 (100%)	47 (100.0%)	103 (100.0%)	31 (100.0%)
Tacrolimus	163 (90.1%)	39 (83.0%)	93 (90.3%)	31 (100.0%)
Mycophenolate mofetil	158 (87.3%)	46 (97.9%)	89 (86.4%)	23 (74.2%)

a Data are the mean±SD or n (%) as appropriate. BMI, body mass index; COPD, chronic obstructive pulmonary disease; ILD, interstitial lung disease; CMV, cytomegalovirus; TMP/SMX, trimethoprim-sulfamethoxazole.

b At sampling.

c Positive bacterial culture could be due to the presence of respiratory pathogens or colonized bacteria. If there was no clear clinical evidence for respiratory infection or no previous culture for reference, the microorganisms in sputum were defined as colonized bacteria.

Clinical characteristics, such as laboratory parameters, hospital stay, intensive care unit (ICU) stay and pulmonary function, are regarded as factors that influence both clinical diagnosis and the airway microbiota. To assess the contribution of clinical variables to microbial community composition and clinical diagnosis, redundancy analysis (RDA) was performed at the operational taxonomic unit (OTU) level. The results showed that the event-free, infection and rejection groups could be distinguished from each other (all *P < *0.05). Several clinical characteristics, such as hospital stay and ICU stay, were highly positively associated with the airway microbiota of infection recipients (Fig. S1a).

Then, we performed a Spearman correlation analysis between the most abundant bacterial genera (relative abundance >1% in at least one group) and the clinical characteristics of LTRs (Table S1). The results showed that the airway microbiota was closely associated with the clinical characteristics of LTRs. For example, *Stenotrophomonas* was positively correlated with invasive mechanical ventilation (IMV) duration and sequential organ failure assessment (SOFA) score, while Haemophilus and *Neisseria* were significantly negatively correlated with IMV duration and SOFA score, suggesting possible harmful or beneficial effects of these airway microbiota on LTRs. Furthermore, we built correlation networks to demonstrate the interaction between the airway microbiota in different groups (Fig. S2). A total of 61, 98 and 75 interactions were identified in the cooccurrence network of the event-free, infection and rejection recipients, respectively. Among them, there were 81.2%, 64.3% and 49.3% positive correlations in the 3 groups, respectively. The networks indicated complex microbial correlations in LTRs, as well as a stronger correlation among the airway microbiota in LTRs with complications than in clinically stable LTRs.

### The airway microbial community in LTRs with different transplant outcomes.

**FIG 2:**
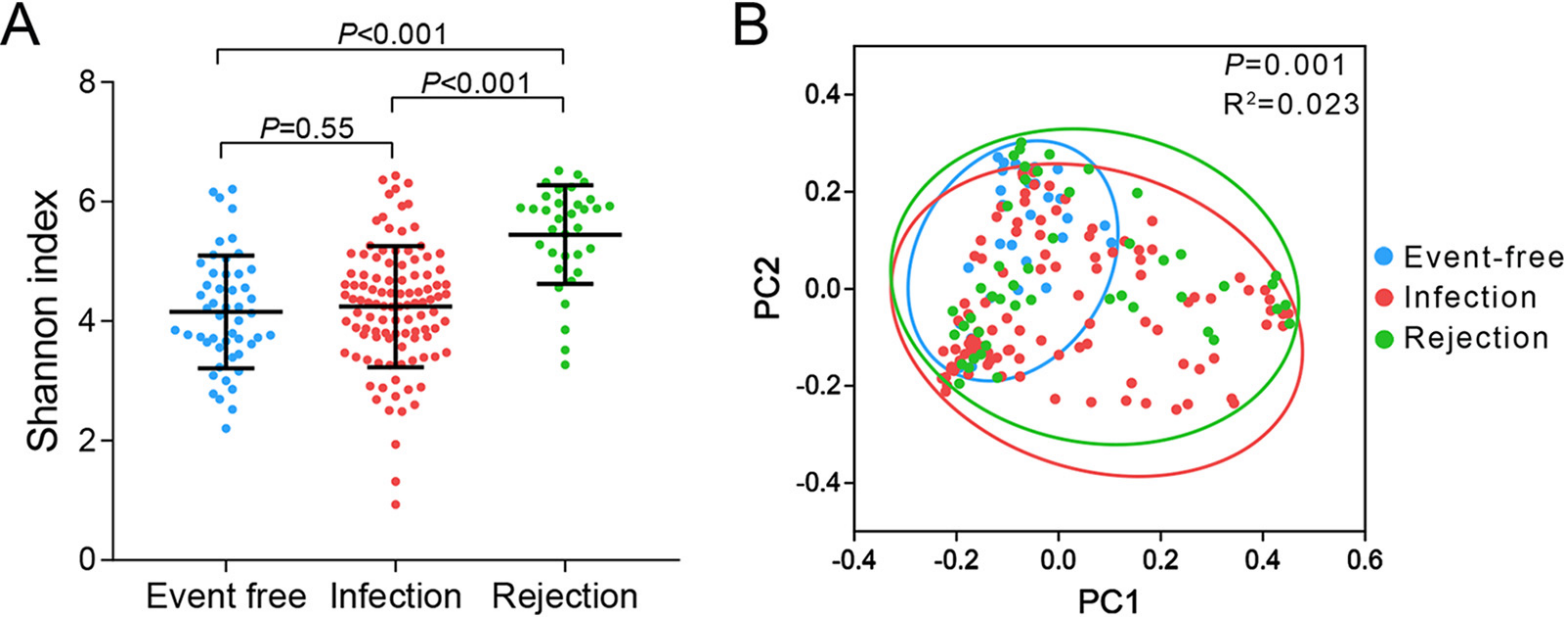
Airway microbial diversity of sputum samples in the event-free, infection and rejection recipients. (A) Alpha diversity (Shannon index) among the 3 transplant groups. The horizontal lines in the box plots represent median values; upper and lower ranges of the box represent the 75% and 25% quartiles. *P* values are represented using the Wilcoxon rank-sum test. (B) Beta diversity (PCoA-based unweighted UniFrac distance matrix) among the 3 transplant groups.

First, we compared the airway microbial diversity among LTRs with different clinical diagnoses. Alpha diversity was significantly different between the event-free and rejection groups and between the infection and rejection groups (both *P < *0.001), with the highest Shannon index in the rejection group (Fig. 2A).

Principal coordinate analysis (PCoA) based on the unweighted UniFrac distance matrix showed distinct beta diversity among the 3 transplant groups (*P = *0.001, R^2^ = 0.023, Fig. 2B), as well as between the event-free and rejection groups and between the infection and rejection groups (*P = *0.002, R^2^ = 0.038; *P = *0.001, R^2^ = 0.022, respectively, Fig. S1c-d). However, the event-free and infection groups were not clearly separated in alpha (Shannon, *P = *0.55, Fig. 2A) and beta diversity (unweighted UniFrac, *P = *0.396, R^2^ = 0.007, Fig. S1b).

**FIG 3:**
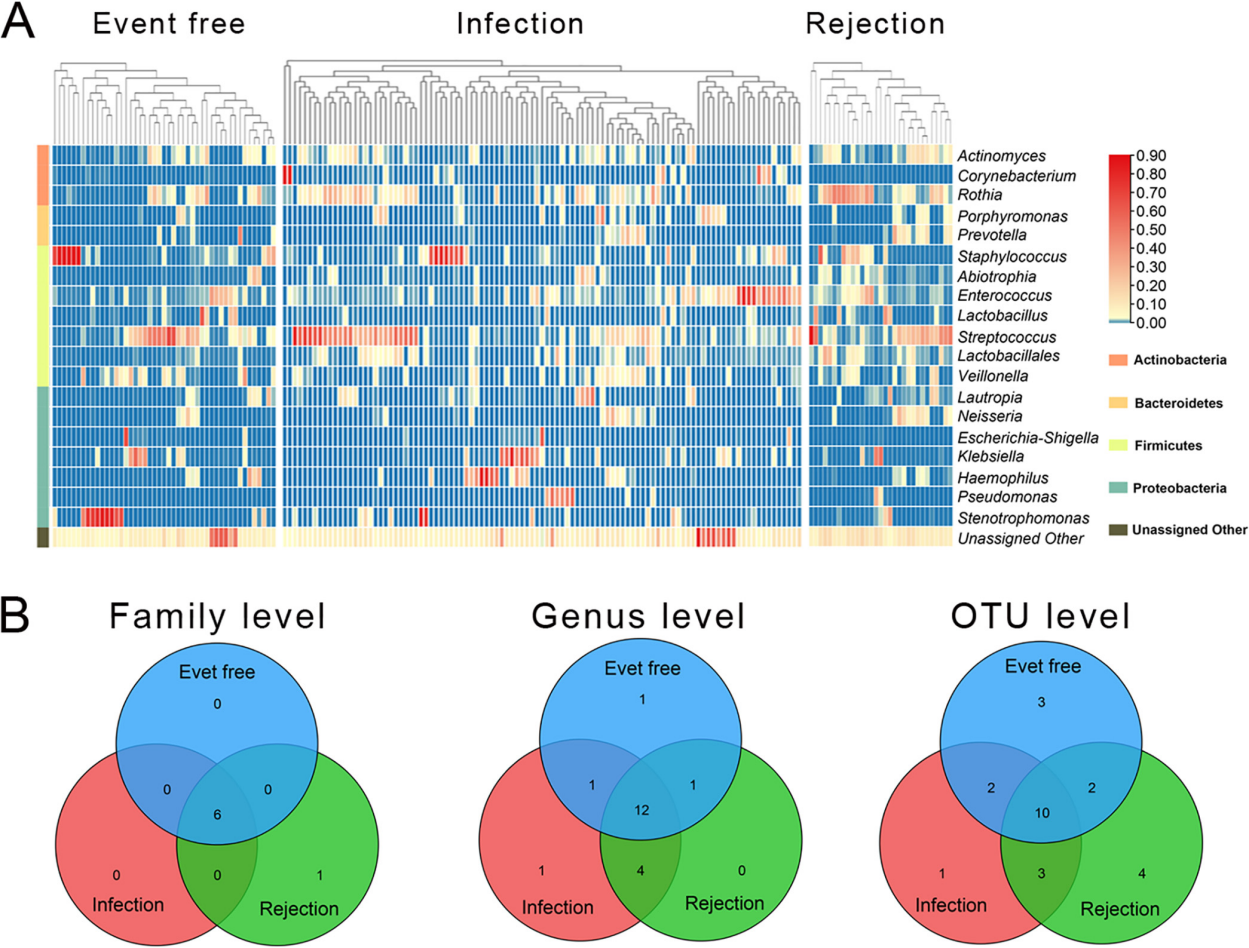
The airway microbial composition of sputum samples in the event-free, infection and rejection recipients. (A) Heat map of the 23 dominant genera (with average relative abundances >1% in at least 1 group) in the event-free, infection and rejection groups. (B) The Venn diagram demonstrates the unique and shared airway microbial numbers at the family level, genus level and OTU level among the different transplant groups.

Additionally, a heat map of the 20 dominant genera (relative abundance >1% in at least one group) showed a different microbial profile among the 3 groups (Fig. 3A). A Venn plot was drawn at the family, genus, and OTU levels with relative abundance >1% among the different groups (Fig. 3B). The results showed that 6 families, 12 genera and 10 OTUs were shared by the event-free, infection and rejection recipients. Several microbial taxa were unique to the 3 groups, including 0 family, 1 genus and 3 OTUs in the event-free group; 0 family, 1 genus and 0 OTU in the infection group; and 1 family, 0 genus and 4 OTUs in the rejection group. This finding indicated that the 3 groups not only shared a common microbiota but also had their own unique taxa which may be associated with the pathogenesis of both diseases.

### Changes in the airway microbiota during infection and rejection of LTRs.

**FIG 4:**
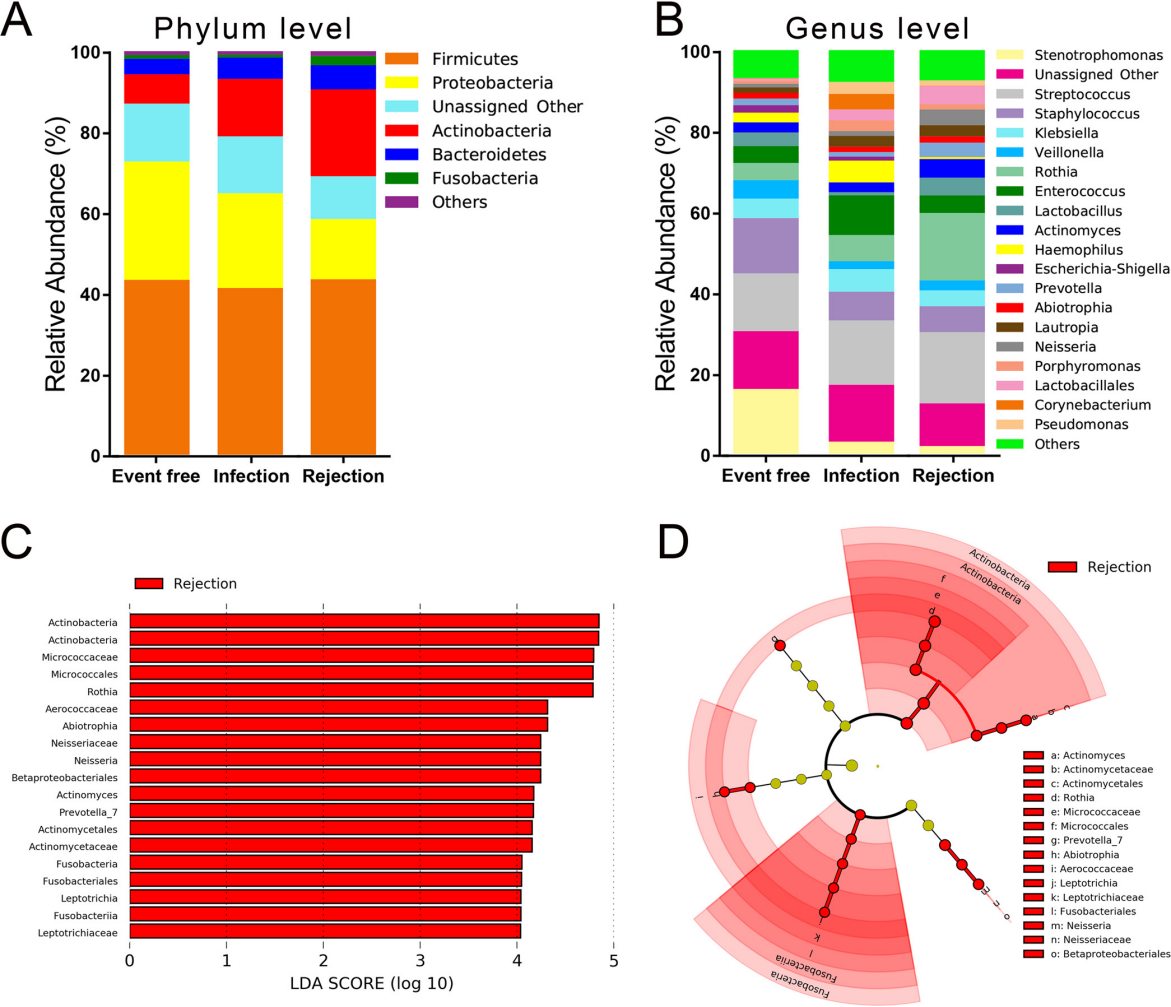
The airway microbial differences among sputum samples collected from the event-free, infection and rejection recipients. Comparison of the relative abundance of airway microbiota based on the most abundant phyla (A) and genera (B) (average relative abundances >1% in at least one group) among LTRs with different clinical diagnoses. Histogram of LDA scores (C) and LEfSe cladogram (D) for differentially abundant bacterial taxa among the 3 groups.

The top 5 most abundant phyla of the airway microbiota detected in our study were Firmicutes, Proteobacteria, Actinobacteria, Bacteroidetes and Fusobacteria. The 20 dominant genera with an average relative abundance greater than 1% in at least one group (mainly *Stenotrophomonas*, Streptococcus, Staphylococcus, Klebsiella, *Veillonella*, and *Rothia*) accounted for up to 90% of the total genera. The microbial composition of the airway microbiota at the phylum level and the genus level was distinct among the event-free, infection and rejection recipients (Fig. 4A and B).

Moreover, we used LEfSe analysis to identify the airway microbiota that were differentially altered among the 3 transplant groups. Only those taxa with LDA scores >4.0 were ultimately considered, and a total of 19 differential taxa were identified (Fig. 4C and D). Among them, 6 bacterial genera, namely, *Actinomyces* (belonging to the phylum Actinobacteria and family Actinomycetaceae), *Rothia* (belonging to the family Micrococcaceae), *Abiotrophia* (belonging to the family Aerococcaceae), *Neisseria* (belonging to the family Neisseriaceae), *Prevotella*, and *Leptotrichia* (belonging to the family Leptotrichiaceae) were greatly enriched in the rejection group, while no differentially abundant genera were found in clinically stable recipients or patients with infection. Table S2 compares the relative abundance and prevalence of the 6 bacterial genera between the 3 groups.

### The prediction efficiency of the airway microbiota and clinical features for different clinical diagnoses of LTRs.

**FIG 5:**
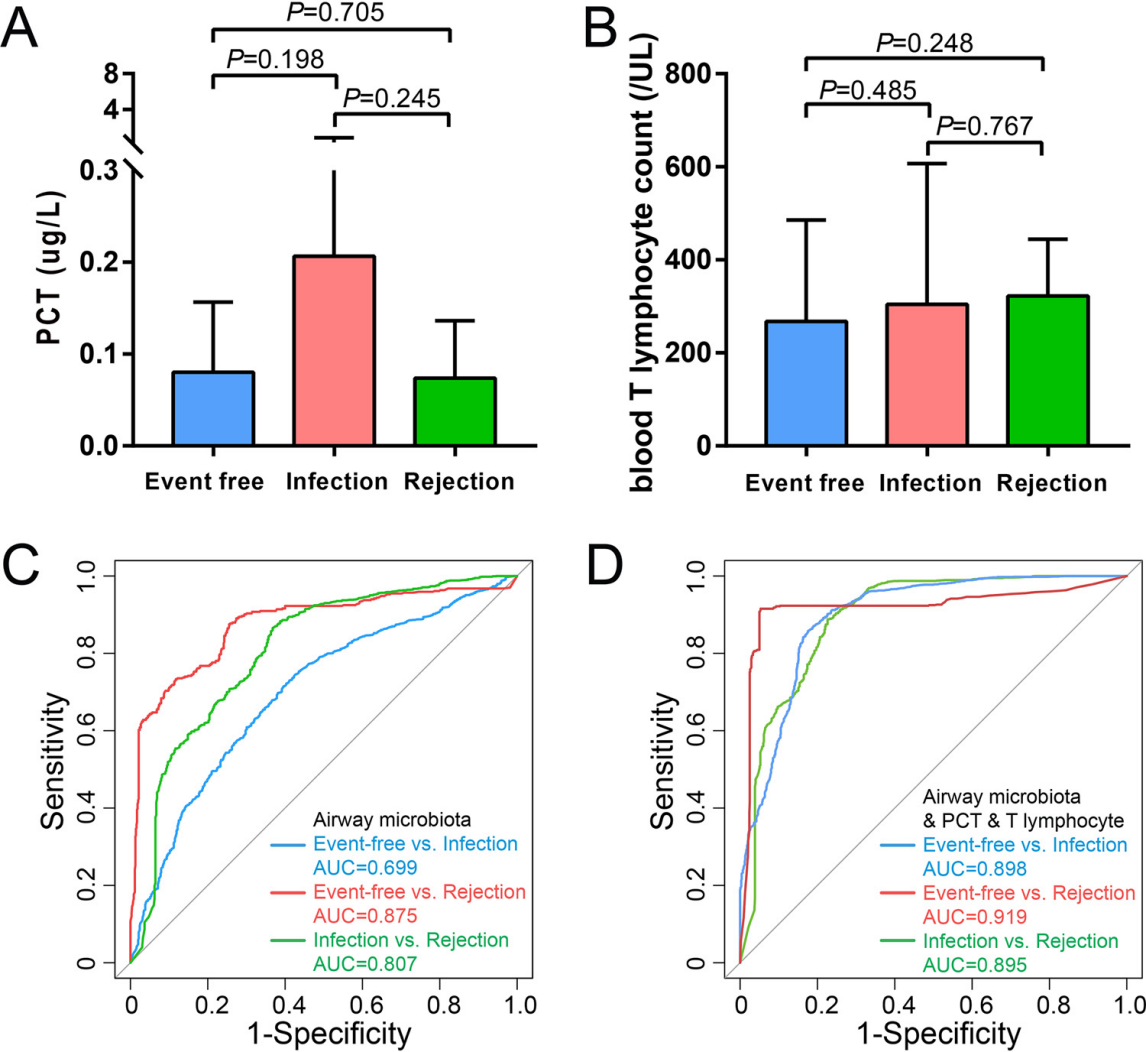
The relationship between clinical variables, airway microbiota and the diagnosis of LTRs. Comparison of (A) PCT and (B) T lymphocyte levels among transplant groups. *P* values are represented using the *t* test. The ROC curve using (C) the airway microbiota alone and (D) the combination of the airway microbiota and PCT and T lymphocyte levels to differentiate among different transplant groups.

Finally, we attempted to evaluate whether there were useful adjunctive indicators for the discrimination of different transplant groups. Generally, increases in serum PCT and peripheral blood T-lymphocyte levels are associated with infection and acute rejection after LT, respectively (20, 21). In our study, PCT and T-lymphocyte levels were relatively high in the recipients with infection and rejection, respectively, but the differences were not statistically significant (Fig. 5A and B). Therefore, random forest analysis was performed using individual airway microbiota constituents alone or in combination with clinical variables (PCT and T-lymphocyte levels) to investigate their prediction efficiencies in LTRs with different clinical diagnoses. First, the receiver operating characteristic (ROC) curve was determined using the relative abundance of the above 6 bacterial genera identified by LEfSe, with AUC values of 0.699 (95%CI: 67.63–72.24%), 0.875 (95%CI: 85.28–89.77%) and 0.807 (95%CI: 78.24–83.10%) to distinguish between the event-free and infection, event-free and rejection, and infection and rejection groups, respectively (Fig. 5C). In comparison, the model was built based on the combination of the 6 bacterial genera and PCT and T lymphocyte levels. The results revealed an improved performance, and the corresponding AUCs reached 0.898 (95% CI: 88.50–91.00%), 0.919 (95% CI: 90.20–93.68%) and 0.895 (95% CI: 87.83–91.10%, Fig. 5D). These results indicated that the airway microbiota, especially combined with PCT and T lymphocyte levels, was a reliable indicator of infection and acute rejection in LTRs.

## DISCUSSION

In this study, we explored the relationship between airway microbiota and infection and acute rejection of LTRs. A significantly different airway microbiota was observed among event-free, infection and rejection recipients. The 6 most differentially genera found by LEfSe analysis were considered to be potential indicators for acute rejection diagnosis in LTRs, including *Actinomyces*, *Rothia*, *Abiotrophia*, *Neisseria*, *Prevotella*, and *Leptotrichia*. Furthermore, a combination of the 6 genera and PCT and T lymphocyte levels indicated great discrimination for different clinical diagnosis, suggesting that the airway microbiota may be a useful indicator for infection and acute rejection diagnosis in LTRs.

Previous studies have found lower microbial richness and diversity but a higher bacterial burden in the lungs of LTRs than in those of control subjects (7), as well as a similar alpha diversity and a distinct beta diversity in the airways before and after LT (8). However, few studies have reported microbial differences among clinically stable, infection, and rejection LTRs. In this study, we found different alpha and beta diversities between event-free and rejection groups and between infection and rejection groups, but no marked difference in either alpha or beta diversity was found between event-free and infection recipients. Venn plots indicated both similarities and differences among the 3 transplant groups. In addition, our results showed close relationship between airway microbiota and clinical characteristics as well as complex microbial interactions in the airways of LTRs. Microbiota composition analysis revealed microbial changes following different diagnoses of LTRs. Moreover, LEfSe analysis identified 19 bacterial taxa (including 6 genera) that were specifically enriched in rejection recipients, suggesting a close association between the airway microbiota and infection and acute rejection in LTRs.

Among the 6 most differential genera, *Actinomyces*, *Rothia*, *Neisseria* and *Leptotrichia* have been found to be associated with asthma (22–27). The increased abundance of *Prevotella* was reported to be associated with T helper type 17 (Th17) immune responses in the lung (28), suggesting an interaction between the 5 microbiota and the immune system in the lung. In addition, *Abiotrophia* were enriched in the sputum of lung cancer cases compared with controls (29), its relationship with the immune system or lung transplantation is unclear. In the current study, the above 6 bacterial genera were enriched in recipients with acute rejection, indicating that they may be involved in the immune response in acute rejection. However, few studies have reported the role of these 6 bacterial genera in allograft lung rejection, and the mechanism remains unknown.

Infection and rejection are very common complications associated with increased morbidity and mortality following LT (13). Unfortunately, the differential diagnosis of infection and rejection is sometimes difficult due to similar clinical manifestations (18, 30). Several studies have suggested that upregulated PCT levels reflect the presence of infective complications after solid organ transplantation and LT (20, 30). T-lymphocyte is regarded as the major immune lymphocytes responsible for lung allograft rejection (21, 31, 32). An increase in peripheral blood T-lymphocyte counts was observed during acute lung rejection (33). However, in our results, infection and rejection recipients showed only a slight increase in PCT and T lymphocyte levels compared with those in the other 2 groups (*P > *0.05). Therefore, we attempted to seek another indicator to differentiate between infection and rejection. We performed random forest analyses and achieved a satisfactory prediction effect of the airway microbiota, especially with the above 6 differential genera identified by LEfSe (*Actinomyces*, *Rothia*, *Abiotrophia*, *Neisseria*, *Prevotella*, and *Leptotrichia*), for distinguishing between different clinical diagnoses (event-free versus infection, AUC = 0.699; event-free versus rejection, AUC = 0.875; infection versus rejection, AUC = 0.807). Furthermore, a better classification efficacy of the combination of the 6 airway microbiota and PCT and T lymphocyte levels was assessed: event-free versus infection, AUC = 0.898; event-free versus rejection AUC = 0.919; and infection versus rejection, AUC = 0.895. Overall, these findings further confirmed the importance of the 6 airway microbiota constituents in transplant infection and rejection and provided important evidence that the airway microbiota was potentially helpful in predicting infection and acute rejection in LTRs. Nevertheless, biomarker‐based models still need to be confirmed with larger samples.

Notably, our study compared the airway microbial profiles between recipients with infection and rejection after LT, as well as evaluated the use of airway microbiota in differentiating between LTRs with infection and rejection. Another strength of this study is a relatively large number of LTRs and the first exploration of the airway microbiota in Chinese LTRs using high-throughput technology (12, 34). A major limitation is the cross-sectional design that provides only the possible relationships between the airway microbiota and infection and rejection in LTRs. Larger studies are needed to repeat and confirm these findings, and future *in vivo* and *in vitro* experiments are needed to determine the microbial-mediated mechanism. The dynamic changes in the airway microbiota may be more reliable and meaningful. In addition, another limitation is that the participants provided different numbers of samples to the study.

In summary, our study explored the relationship between the airway microbiota and LTRs with infection and acute rejection. *Actinomyces*, *Rothia*, *Abiotrophia*, *Neisseria*, *Prevotella*, and *Leptotrichia* were enriched in recipients with acute rejection. The combination of airway microbiota with PCT and T-lymphocyte levels may complement the deficiencies of the diagnosis of acute rejection and infection in LTRs and thus improve the treatment and clinical outcomes of patients after LT. In the future, it seems important to understand the detailed role of the airway microbiota in the mechanism and development of infection and acute rejection after LT.

## MATERIALS AND METHODS

### Study design and subjects.

A total of 181 sputum samples collected from 59 adult LTRs were enrolled in our study at the First Affiliated Hospital of Guangzhou Medical University (Guangzhou, China) between June 2017 and December 2019. This study was approved by the ethics committee of the First Affiliated Hospital of Guangzhou Medical University (no 2017-22). All patients provided written informed consent, in accordance with the Declaration of Helsinki. Clinical information collected from the recipients included standardized medical record abstraction, including the demographic data, transplant data, laboratory examination and clinical diagnosis of the recipients (Table 1).

Adult LTRs (age >18 years) hospitalized in the First Affiliated Hospital of Guangzhou Medical University from June 2017 to December 2019 were included. The exclusion criteria included intubation or mechanical ventilation, or other complications (e.g., bleeding, anastomotic complications, pneumothorax, etc.), or undetermined diagnoses or the coexistence of infection and rejection at the time of sampling.

The diagnostic criteria for respiratory infection were based on clinical (such as fever, cough, sputum production and radiographic infiltrate) and microbiological grounds (14, 35, 36), and are summarized as follows:

(i) Signs/symptoms: at least one of the following: (a) fever >38°C or hypothermia <36.5°C with no other recognized source; (b) leukocyte count <4000 or >15000/mm^3^; (c) purulent secretions; (d) new onset or worsening cough, dyspnea, tachypnea or plural rub, rales, or bronchial breath sounds; (e) worsening gas exchange (O_2_ desaturation, PaO_2_/FiO_2_ < 240) increasing the O_2_ requirement andthe ventilation demand; and (f) pleural effusion.
(ii) Radiology: new/worsening radiographic infiltrate on chest X-ray or CT scan.
(iii) Microbiology: at least one of the following: (a) positive growth in blood culture unrelated to other sources; (b) positive growth of pleural fluid; (c) positive respiratory culture (sputum, bronchial secretions, BALF, or bronchial sterile brushing); and (d) >5% of BALF-obtained cells contained intracellular bacteria on direct microscopic examination.

As a supplement, markers such as neutrophil proportion and PCT may help in the diagnosis of infection (37, 38). It is noteworthy that positive bacterial culture could be due to the presence of respiratory pathogens or colonized bacteria. In LTRs with a positive culture, those who did not fulfill the clinical criteria for respiratory infection (lack of symptoms/signs and radiologic changes) were classified by 2 clinicians as colonized patients (39). Thereafter the recipients with colonization were divided into groups according to the criteria of rejection and event-free. In addition, if fungal infection was suspected, other tests such as galactomannan, 1,3‐β‐D‐glucan assay or even PCR, could be performed, although their efficacy is limited (2). Finally, for a diagnosis of bacterial infection, LTRs who were infected with only fungi or viruses were excluded.

Acute allograft rejection presents with nonspecific features, such as shortness of breath, cough with or without sputum production and even low-grade fever. According to the Heart and Lung Transplantation (ISHLT) criteria, pathological findings in transbronchial biopsy specimens are the gold standard for the diagnosis of acute rejection after LT (40). The diagnosis of acute rejection is based on perivascular and interstitial mononuclear infiltrates. Moreover, the cytology of BALF and peripheral blood, such as eosinophil count, lymphocyte count and basophil count, may suggest a tendency to acute rejection (41).

Clinically stable recipients (or event-free) were either discharged to common ward from the ICU or hospitalized for reexamination and were defined as having neither infection nor rejection. Each sample was assigned a diagnosis of infection, acute rejection or event-free independently by two experienced clinicians. In the case of disagreement, a third clinician was consulted.

In addition, 11 recipients were continually included into our study, and they were sampled in two clinical statuses (same or different) at different time periods. Among the 11 recipients, one recipient was grouped to the event-free group, and five recipients were divided into the infection group at two sampling periods. In addition, one recipient was diagnosed with event-free and rejection; two recipients were diagnosed with event-free and infection; and two recipients were diagnosed with infection and rejection in two sampling periods (Fig. 1).

### Data collection.

For each enrolled subject, we collected demographic data, operation-related data, laboratory tests and pharmacological treatment. Except for operation-related data collected during or after LT, other data were collected at the time of sampling. Laboratory data, including cell counts/percentages in the blood and BALF, the biomarkers of inflammation (e.g., PCT) and microbiological data, were provided by the clinical laboratory of the First Affiliated Hospital of Guangzhou Medical University. The absolute count of total T lymphocytes (CD3^+^) in peripheral blood was determined by flow cytometry using the Cytomics FC500 cytometer. Serum PCT was measured using an electrochemical luminescence immunoassay. PGD was diagnosed and graded based on pulmonary edema on chest X-ray and the PaO_2_/FiO_2_ ratio according to the 2016 ISHLT consensus statement (42).

### Sample collection, DNA extraction and 16S rRNA gene sequencing.

Sputum samples were obtained by induced sputum of hypertonic or isotonic saline with salbutamol according to the Task Force on Induced Sputum of the European Respiratory Society (43). All samples were immediately stored at −80°C for subsequent DNA extraction. Frozen sputum samples were thawed and centrifuged at 10,000 × *g* for 3 min. The supernatant was discarded and genomic DNA extraction was performed using a Bacterial DNA Extraction minikit (Mabio, Guangzhou, China) as follows. (1) 30 μL Lysozyme, 220 μL Buffer STE and 5 μL RNase Solution were added, and the mixture was placed in a water bath at 37°C for 10 min. (2) Then, 250 μL Buffer MBL and 20 μL Proteinase K were added to the lysis solution, and the mixture was briefly mixed on a vortex mixer and then shaken for 10 min in a warm bath at 70°C. (3) A total of 250 μL absolute ethanol were added to the supernatant, then vortexed and mixed for 15 s. (4) DNA Extraction Mini Columns I was put into a 2 mL collecting tube. The mixture obtained in the third step was added to the column, which was centrifuged at 10,000 × *g* for 1 min. (5) The centrifuged effluent discarded, and the column was put into the collecting tube. Then, 600 μL Buffer W1A (diluted with ethanol) was added to the column, which was centrifuged at 10,000 × *g* for 10 s. (6) The filtrate was discarded after centrifugation, and the column was placed into the collecting tube. Then, 600 μL Buffer W2A (diluted with ethanol) was added to the column, which was centrifuged at 10,000 × *g* for 10 s. (7) The centrifuged effluent was discarded, and the column was placed into the collecting tube. (8) Steps 6–7 were repeated, and the column was centrifuged at 13,000 × *g* for 2 min. (9) The column was placed in a new 1.5 mL centrifuge tube, then 30–150 μL Buffer EB preheated to 65°C was added to the center of the column membrane, which was incubated for 3 min and then centrifuged at 13,000 × *g* for 1 min. (10) The DNA binding column was discarded, and the DNA was stored at 2–8°C. For long-term storage, the DNA should be stored at −20°C.

DNA was quantified with a NanoDrop One (Thermo Scientific, USA). PCR amplification was performed using specific primers with barcodes and TaKaRa Premix Taq Version 2.0 (TaKaRa Biotechnology Co., Dalian, China), using genomic DNA as a template. The V3-V4 hypervariable region of the 16S rRNA gene was amplified by PCR with the forward primer: 338F 5′-ACTCCTACGGGAGGCAGCA -3′ and reverse primer: 806R 5′-GGACTACHVGGGTWTCTAAT-3′. PCR amplification was performed following the cycling protocol (Bio-Rad S1000 [Bio-Rad Laboratory, CA]) initial denaturation step at 94°C for 5 min; 30 cycles at 94°C for 30 s, 52°C for 30 s and 72°C for 30 s; and a final extension at 72°C for 10 min. Three replicates were performed for each sample, and PCR products from the same sample were mixed. The amplification reaction system included 50 ng of genomic DNA template; 25 μL of 2× Premix Taq; 1 μL of 10 μmol L^-1 primer 338F; 1 μL of 10 μmol L^-1 primer 806R; and nuclease-free water added to 50 μL. The PCR products were detected using 1% agarose gel electrophoresis. All the amplification products were stored at −80°C for subsequent sequencing.

The amplicons were sequenced using the Illumina Hiseq 2500 platform (Guangzhou, China). Full details about the DNA amplification, purification and preparation for sequencing are provided in our previous study (44, 45). Subsequent sequence processing and analysis were performed using the Quantitative Insights into Microbial Ecology (QIIME) platform (46). First, the barcode primers were trimmed and filtered if they contained ambiguous reads or mismatches in the primer regions following the barcoded Illumina paired-end sequencing (BIPES) protocol (47). Next, we screened and removed chimeras using UCHIME in *de novo* mode to obtain high-quality sequence reads of the 16S rRNA gene (48). Eight samples were excluded from the 16S V3-V4 data analysis after normalization. The taxonomy of representative 16S rRNA gene sequences were classified using the Silva 132 database, and multiple alignments of representative sequences were performed using Python Nearest Alignment Space Termination (PyNAST) (49). Representative 16S rRNA gene sequences were classified into specific taxa using the Ribosomal Database Project (RDP) classifier (50). The OTUs were assigned by clustering the reads with 97% sequence similarity using USEARCH (51). Contamination sequences were removed from the sequences, and all samples were normalized to 28,000 sequences to avoid deviation caused by the effects of different sequencing depths.

### Statistical analysis.

The Shannon index was used to evaluate the alpha diversity (within-sample diversity). PCoA based on the unweighted UniFrac distance matrix was performed to calculate beta diversity (dissimilarity between samples). Differentially abundant bacterial taxa among groups were identified using LEfSe analysis with a threshold set at 4.0 for the LDA score (52). RDA was performed to evaluate the relationship between clinical characteristics and different groups. Spearman correlation analysis was performed to evaluate the relationship between the airway microbiota and clinical characteristics of LTRs. Network analysis using Spearman rank correlation was performed using Cytoscape (v3.7.2), and only significant associations with *P* values <0.05 after false discovery rate (FDR) correction were included. Random forest models were used to classify the LTRs with different diagnoses and evaluate the importance of indicator using the R “randomForest” package (53). The performance of the model was evaluated using a 10-fold cross-validation approach and measured using a ROC curve (“pROC” package). The AUC was determined to assess the ROC effect. Clinical characteristics were evaluated using SPSS (v20.0) software, and Figures were generated using GraphPad Prism (v7.0), Canoco (v5.0), TBtools (v1.09832), and R (v2.1.1) software.

### Data availability.

The raw sequencing data were deposited in ENA (accession number PRJEB40386).
