# Supplementary material for: The Airway Microbiota Signatures of Infection and Rejection in Lung Transplant Recipients
Source: Microbiol Spectr. 2022 Apr 13;10(2):e00344-21. doi: 10.1128/spectrum.00344-21 (PMC9045364; doi:10.1128/spectrum.00344-21)
Supplement: SUPPLEMENTAL FILE 1 — Supplemental material. Download spectrum.00344-21-s0001.pdf, PDF file, 0.6 MB [file spectrum.00344-21-s0001.pdf]

# Supplemental Materials

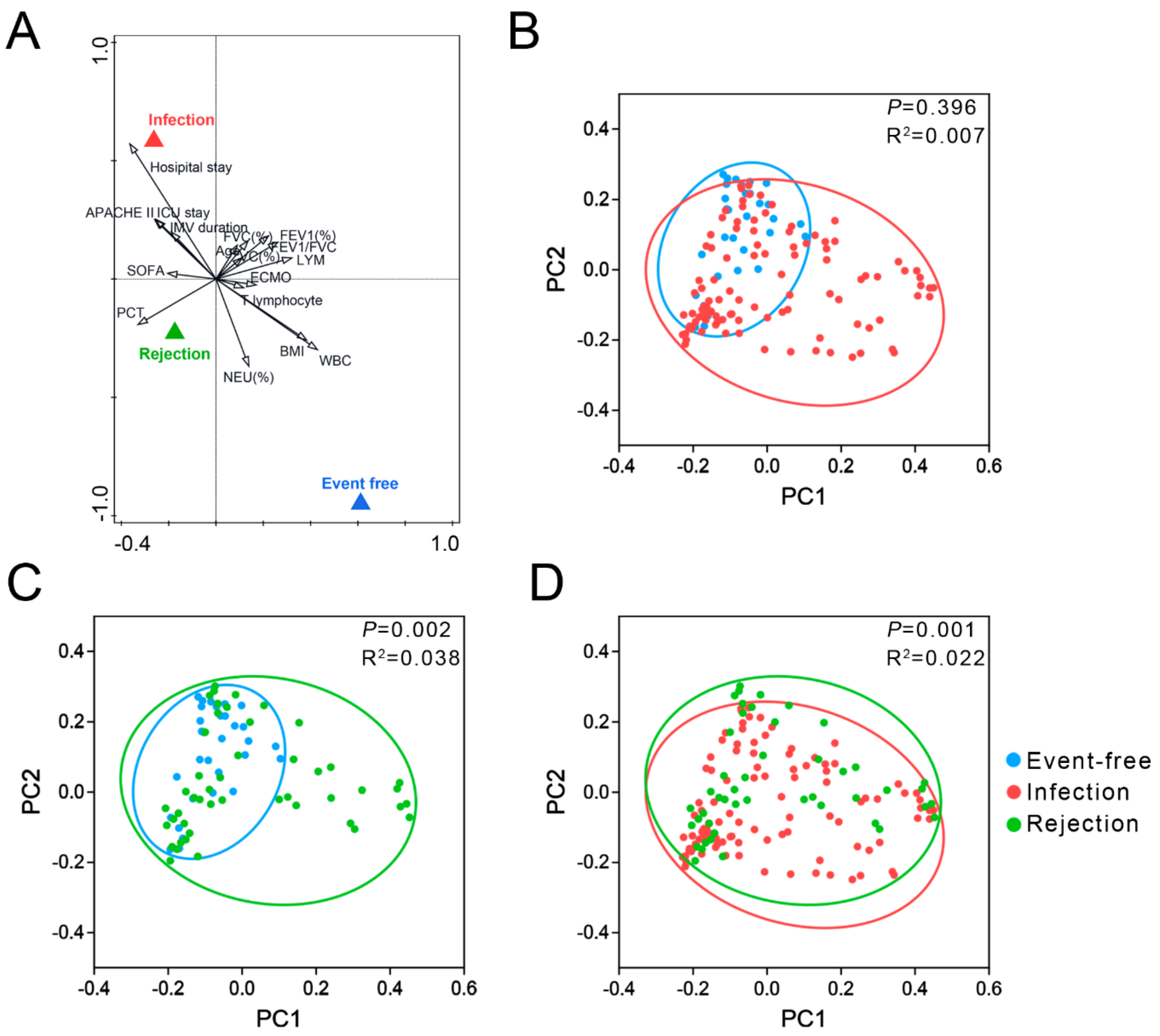


**FIG S1.** (A) RDA shows the relationship between the airway microbiota and clinical variables of LTRs. The solid triangle represents the clinical diagnosis, and the black arrow represents a clinical variable. The clinical variables with nonobvious associations with any one of the diagnoses (*P*>0.05) are not presented. Abbreviations: APACHE, acute physiology and chronic health evaluation; BMI, body mass index; ECMO, extracorporeal membrane oxygenation; FEV1, forced expiratory volume in the first second; FVC, forced vital capacity; NEU, blood neutrophils; IMV, invasive mechanical ventilation; LYM, blood lymphocyte; SOFA, sequential organ failure assessment; VC, vital capacity; WBC, white blood cell; (B-D) Beta diversity (PCoA-based unweighted UniFrac distance matrix) between each 2

groups. The blue, red and green colors represent the event-free, infection and rejection groups, respectively.

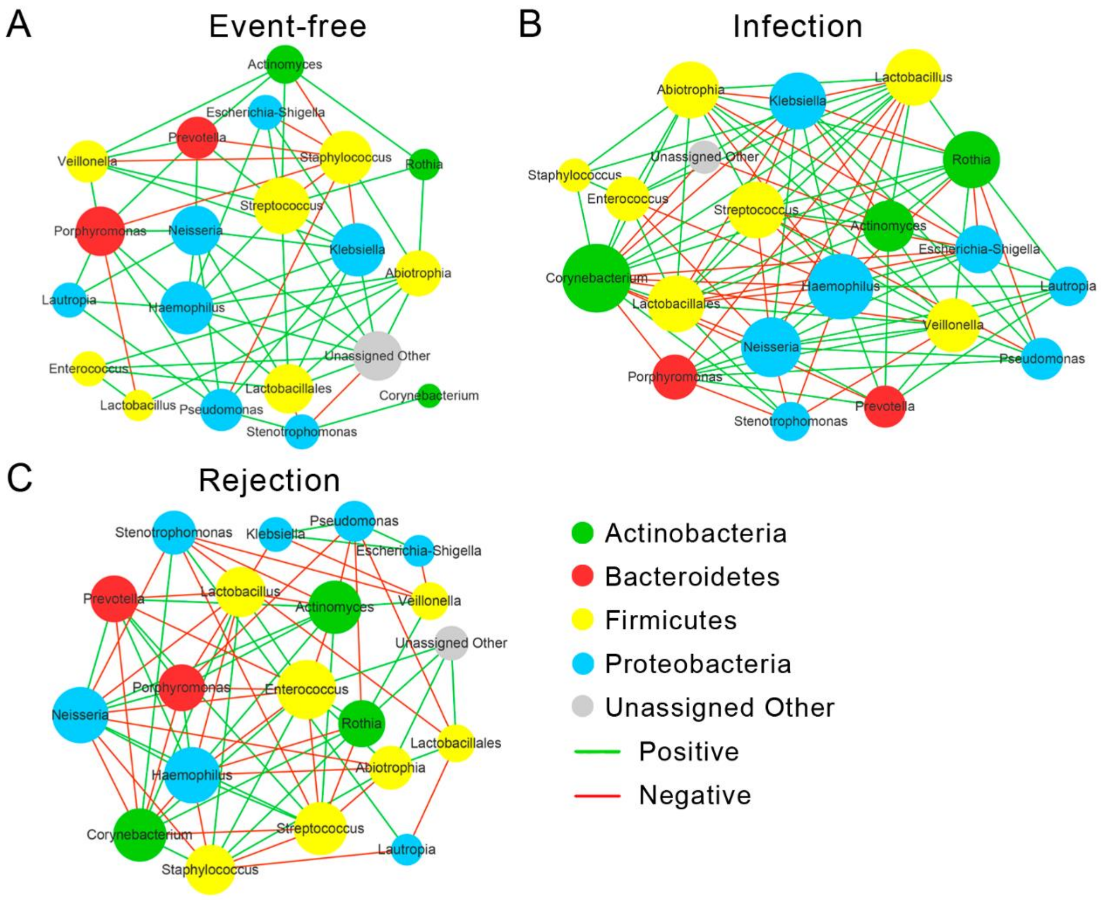


FIG S2. Co-occurrence networks of the airway microbiota in different groups. Networks based on the most abundant genera (average relative abundances >1% in at least one group) in event-free (A), infection (B) and rejection (C) recipients. Each node represents a microbial taxon and is colored by phyla. The node size represents the degree of connectivity. Green and red edges represent positive and negative correlations, respectively (determined using Spearman rank correlation tests). Only significant correlations with FDR P-values<0.05 are displayed.

**TABLE S1.** Correlation between the airway microbiota and the clinical characteristics of LTRs.

| Genus | Hospital stay | IMV duration | APACHE II score | SOFA score | T lymphocytes | WBC | LYM | NEU | FEV1 (%) | FEV1/FVC (%) |
|---|---|---|---|---|---|---|---|---|---|---|
| *Stenotrophomonas* | | + | | + | | | | | | |
| *Unassigned Other* | | | | | | | | | | |
| *Streptococcus* | | | | | | - | | | | - |
| *Staphylococcus* | | | | | | | | | + | |
| *Klebsiella* | | | | | | | | | | |
| *Veillonella* | | | | | | | | | | |
| *Rothia* | | | - | | | | | | | |
| *Enterococcus* | | | | | | | | | | |
| *Lactobacillus* | | | | + | | | | | | |
| *Actinomyces* | | | - | | | | | | | |
| *Haemophilus* | | - | | - | | | | + | | |
| *Escherichia-* | | | | | | | | | | |
| *Prevotella* | | | | | | | | | | |
| *Abiotrophia* | | | | | | - | | | | |
| *Lautropia* | | | | | + | | | + | | |
| *Neisseria* | | - | | - | + | | - | + | | |

Statistical significance was assessed by using Spearman rank correlation, and only significant correlations with FDR $P$ <0.05 are presented. "+" represents a positive correlation and "-" represents a negative correlation.

Abbreviations: IMV, invasive mechanical ventilation; APACHE, acute physiology and chronic health evaluation; SOFA, sequential organ failure assessment; WBC, white blood cell; NEU, blood neutrophils; LYM, blood lymphocyte; FEV1, forced expiratory volume in the first second.

**TABLE S2.** The relative abundance (RA%) and prevalence (P%) of the 6 bacterial genera identified by LEfSe among the 3 groups.

| Genus | Event-free | | Infection | | Rejection | | *P* value (RA% between groups) | | |
|---|---|---|---|---|---|---|---|---|---|
| | RA% | P% | RA% | P% | RA% | P% | Event-free vs. Infection | Event-free vs. Rejection | Infection vs. Rejection |
| *Rothia* | 4.24 | 100 | 6.49 | 100 | 16.68 | 100 | 0.061 | <0.001 | <0.001 |
| *Actinomyces* | 2.49 | 100 | 2.41 | 100 | 4.61 | 100 | 0.247 | 0.004 | 0.004 |
| *Neisseria* | 0.93 | 91.49 | 1.19 | 85.44 | 3.87 | 93.55 | 0.85 | 0.003 | 0.002 |
| *Prevotella* | 1.63 | 76.60 | 1.11 | 74.76 | 3.48 | 87.10 | 0.45 | 0.075 | 0.01 |
| *Abiotrophia* | 1.44 | 100 | 1.33 | 100 | 1.58 | 100 | 0.002 | <0.001 | <0.001 |
| *Leptotrichia* | 1.38 | 70.21 | 0.62 | 58.25 | 0.02 | 35.48 | 0.052 | 0.001 | 0.013 |

*P* values are represented using the Wilcoxon rank-sum test.
